# The Key Role of microRNAs in Initiation and Progression of Hepatocellular Carcinoma

**DOI:** 10.3389/fonc.2022.950374

**Published:** 2022-07-18

**Authors:** Suliman Khan, De-Yu Zhang, Ji-Yu Zhang, Mian Khizar Hayat, Jingli Ren, Safyan Nasir, Muhammad Fawad, Qian Bai

**Affiliations:** ^1^ Department of Cerebrovascular Diseases, the Second Affiliated Hospital of Zhengzhou University, Zhengzhou, China; ^2^ Department of Gastroenterology, the First Affiliated Hospital of Zhengzhou University, Zhengzhou, China; ^3^ Ministry of Education (MOE) Key Laboratory of Cell Activities and Stress Adopations, School of Life Sciences, Lanzhou University, Lanzhou, China; ^4^ Zhengzhou Key Laboratory of Big Data Analysis and Application, Henan Academy of Big Data, Zhengzhou University, Zhengzhou, China; ^5^ Allied District Headquarter Hospital, Faisalabad, Pakistan; ^6^ School of Mathematics and Statistics, Zhengzhou University, Zhengzhou, China

**Keywords:** hepatocellular carcinoma, microRNA, oncogene, tumor suppressor, therapy

## Abstract

Hepatocellular carcinoma (HCC) is the main type of primary liver malignancy and the fourth leading cause of cancer-related death worldwide. MicroRNAs (miRNAs), a type of non-coding RNA that regulates gene expression mainly on post-transcriptional level has a confirmed and important role in numerous biological process. By regulating specific target genes, miRNA can act as oncogene or tumor suppressor. Recent evidence has indicated that the deregulation of miR-NAs is closely associated with the clinical pathological features of HCC. However, the precise regulatory mechanism of each miRNA and its targets in HCC has yet to be illuminated. This study demonstrates that both oncogenic and tumor suppressive miRNAs are crucial in the formation and development of HCC. miRNAs influence biological behavior including proliferation, invasion, metastasis and apoptosis by targeting critical genes. Here, we summarize current knowledge about the expression profile and function of miRNAs in HCC and discuss the potential for miRNA-based therapy for HCC.

## Introduction

Hepatocellular carcinoma (HCC) is the fourth leading cause of cancer-related mortality and the seventh most frequent ma-lignancy worldwide (1). HCC accounts for approximately 90% of primary liver cancer, and is considered as one of the deadliest cancers. Infection of hepatitis B or C viruses, alcohol abuse, toxicants and metabolic disorders all increase the risk of liver cancer (2, 3). In addition, ingestion of the fungal metabolite aflatoxin B1, inhalation of smoke, and adeno-associated virus 2 also are associated with an increase in the incidence of HCC. Generally, patients with cirrhosis, and hu-man immunodeficiency virus infection or thalassemia are also at higher risk of HCC (4–6). Ac-cording to e-Medicine-Health, the 5-year survival rate of early stage HCC is 33%, vs. 2%-11% for advanced stage.

Despite the development of various treatment strategies such as antiviral therapies and clinical interventions, the prognosis of HCC remains poor. Thus, there is a need for developing advanced diagnostic and treatment options. During the last decade, researchers have focused on investigating the molecular underpinnings such as genomic aberrations, biomarkers and drug target sites. Among the molecular entities, miRNAs play a crucial role in carcinogenesis and have enormous therapeutic potential, thus gaining serious attention during the last few years (7).

miRNAs regulate the global activity of cells by targeting a range of genes and proteins articulation. Increasing evidence demonstrates that miRNAs assume a key administrative role in Multiple Drug Resistance (MDR). Moreover, miRNAs have been considered as potential biomarkers and therapeutic targets (8).

**Figure 1:**
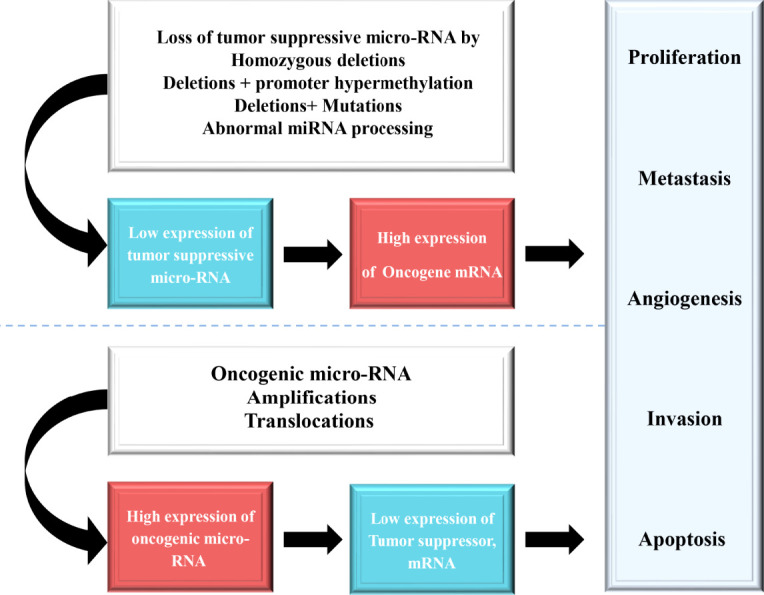
The loss of function of tumor suppressive miRNAs result in an anomalous expression of the target tumor suppressor or oncogene, which contributes to tumor progression.

**Figure 2:**
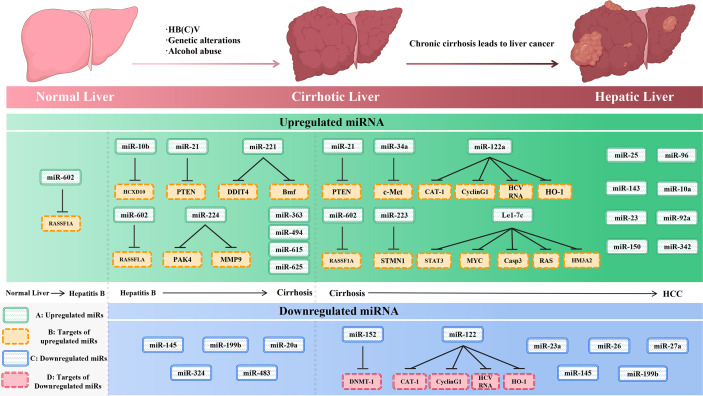
The initiation and development of hepatocellular carcinoma (left) and the expression of miRNAs and their targets (right) during the development of liver cancer. **(A)**-upregulated miR-NAs, **(B)**-targets of upregulated miRNAs, **(C)**-downregulated miRNAs, and **(D)**-targets of downregulated miRNAs.

miRNAs have been found frequently deregulated in HCC and specific miRNAs have been re-ported to be associated with the clinicopathological features including metastasis, recurrence, and prognosis of HCC (9). Moreover, compelling evidence suggested that miRNAs influence HCC development by targeting proliferation, apoptosis and metastasis of tumor cells. The proposed mechanisms by which miRNAs act as oncogenes or tumor suppressor genes were depicted in **Figure 1**. The up-regulated miRNAs and those down-regulated in HCC are shown in **Tables 1** and **2**. **Figure 2** shows a schematic overview of the deregulated miRNAs, their target genes and subsequent phenotypes of the resulting HCCs. The aim of this study is to systemically evaluate differentially expressed miRNAs in HCC patients which have been reported consistently through independent studies. Furthermore, we discuss the clinical applications of miRNAs in HCC diagnosis and treatment strategies.

**Table 1:** Up regulated miRNAs and their target genes in hepatocellular carcinoma.

miRNAs	Potential and Validated targets	Validated targets expressions	Functions of validated targets	References
miR-21	RHOB; PDCD4, RECK, PTEN	Down regulated	Proliferation, survival, invasiveness	(Cao et al., 2019) (10)
miR-27b	FBXW7	Down regulated	Apoptosis, growth arrest	(Sun et al., 2016) (8)
miR-4262	PDCD4	Down regulated	Enhance proliferation, decrease apoptosis.	(Lu et al., 2016) (11)
miR-935	c-Myc, cyclin D1, SOX7,	Down regulated	cell proliferation, cell motility, invasiveness	(Liu et al., 2016) (12)
mir-765	p-FOXO3a, p-AKT p21, INPP4B	Down regulated	cell proliferation, tumorigenicity	(Xie et al., 2016) (13)
miR-519a	cyclin D1, PI3K/Akt, p27, PTEN.	Down regulated	cell proliferation, cell cycle progression, Apoptosis, differentiation	(Tu et al., 2016) (14)
miR-1180	Cyclin D1, Myc, p-Rb, TNIP2.	Down regulated	cell proliferation, cell growth	(Zhou et al., 2016) (15)
miR-761	MFN2	Down regulated	Apoptosis, migration, invasion	(Zhou et al., 2016) (15)
miR-155-3p	FBXW7	Down regulated	cell proliferation, tumorigenesis	(Tang et al., 2016) (15)
miR-135a	MMP2, p-AKT, FOXO1.	Down regulated	Invasion, metastasis, cell migration	(Zeng et al., 2016) (15)
miR-107	CPEB3	Down regulated	cell proliferation, metastasis	(Zhou et al., 2014) (16)
miR-24-3p	SOX7, MT1M	Down regulated	Cell growth	(Dong et al., 2016) (17)
miR-103	PKC _α, AKAP12	Down regulated	Apoptosis, cell proliferation	(Xia et al., 2016) (18)
miR-222	p57, DDIT4, PTEN, Bmf, TIMP3, PPP2R2A, P27	Down regulated	Metastasis, Apoptosis, cell growth	(Yang et al., 2014) (19)

**Table 2:** Down regulated miRNAs and their target genes in hepatocellular carcinoma.

miRNAs	Potential and Validated targets	Validated targets expressions in HCC	Functions of validated targets	References
miR-98	SALL4,	up regulated	proliferation, migration, invasion, chemo resistance	(Zhou et al., 2016) (15)
miR-101	Mcl-1; EED;DNMT3A; SOX9, EZH2	up regulated	proliferation, invasion, colony formation, cell cycle progression, tumorigenesis	(Xu et al., 2013) (20)
MiR-124	PIK3CA,SNAI2, ROCK2, EZH2 ,STAT3	up regulated	proliferation, Apoptosis	(Lu et al., 2013) (21)
miR-133b	GPC3, TAGLN2, SOX9SIRT1	up regulated	proliferation, Apoptosis, invasion, tumorigenesis	(Tian et al., 2016) (22)
miR-502-3P	SET	up regulated	Cell proliferation, Migration, , invasion	(Jin et al., 2016) (23)
miR-613	DCLK1	up regulated	Tumorigenesis, proliferation, invasion	(Wang et al., 2016) (24)
miR-622	NF-κB , JNK, MAP4K4	up regulated	colony formation, proliferation, invasion, Apoptosis	(Song et al., 2015) (25)
miR-449a	POU2F1, FOS , Met ,ADAM10	up regulated	Cell proliferation, colony formation, migration, Invasion. differentiation	(Liu et al., 2016) (12)
miR-144	E2F3 , SMAD4	up regulated	Growth	
miR-634	Rab1A, DHX33	up regulated	cell growth, Colony formation	
miR-625	PTEN, p-Ak , p-HSP27, IGF2BP1	up regulated	Metastasis, Invasion	(Zhou et al., 2016) (15)
miR-26a	STAT3, IL-6	up regulated	Cell survival. Invasion, tumor growth	(Yang et al., 2013) (26)

## Down-Regulated miRNAs in HCC

### miR-26a

miR-26a has been found to be downregulated during the progression and development of HCC. However, its expression level is not connected with tumor size or clinical stage of the HCC. Studies have indicated that overexpression of miR-26a stifles the expression of DNA methyltransferase 3b (DNMT3B), which act as an immediate target of miR-26a, suggesting a tumor suppressive effect of miR-26a. Notably, DNMT3B is significantly upregulated in HCC tis-sues (27, 28).

### miR-98

MiR-98 functions as tumor suppressor or oncogene in different tumor by interacting with various target genes (29). MiR-98 has been found down-regulated in HCC tissues with its expression related to tumor size, metastasis, and portal vein tumor embolus (15). Zhou et al. reported that ectopic expression of miR-98 decreased the proliferation, migration, invasion and epithelial-mesenchymal transition (EMT) of HCC cells by inhibiting SALL4, thus suppress tumor generation. On the contrary, over expression of SALL4 reverses the suppressive effect of miR-98, resulting in accelerated proliferation, migration, invasion and EMT of HCC cells (30).

### miR-101

Reduced expression of miR-101 has been reported in HCC tissues and cell lines (31), especially in HBV related HCC, suggesting a tumor suppressive role of miR-101 in HCC development (32). In addition, overexpression of miR-101 was associated with reduced the expression of DNA me-thyltransferase 3 alpha (DNMT3A) and vice versa. Moreover, miR-101 inhibits the expression of the FBJ murine osteosarcoma viral oncogene homolog (FOS) post-transcriptionally by binding to 3’UTR of FOS mRNA, thereby preventing HCC invasion and migration. Various studies have concluded that miR-101 suppress HCC by targeting an oncogene known as enhancer of zeste homolog 2 (EZH2), myeloid cell leukemia sequence 1 (Mcl-1) and Nemo-like kinase (NLK) [17.18,19]. It is known that targeted disruption of NLK inhibits tumor cell growth by blocking cyclin D1 and CDK2 in human hepatocellular carcinoma (33). These studies have thus confirmed a key regulatory role of miR-101 in HCC progression.

### miR-124

MiR-124 is down-regulated in HCC and is associated with decreased apoptosis and enhanced proliferation. It affects tumor progression with the involvement of STAT3 (34, 35). STAT3 has been reported to be activated significantly in HCC patients with poor prognosis. MiR-124 also suppresses ROCK2 and EZH2 on both the mRNA and protein levels, which in turn inhibits epithelial-mesenchymal cell transition (36). Some reports have also indicated that miR-124 targets PIK3CA to inhibit cell proliferation (35).

### miR-133b

miR-133b mostly acts as a tumor suppressor and is downregulated in HCC suggesting its involvement in cancer progression or development (37). It targets the Sirt1 gene which plays a key role in the development of different cancers. miR-133b if over-expressed, can attenuate cell proliferation and invasion, and increase apoptosis *via* the miR-133b/Sirt1/GPC3/Wnt β-catenin axis pathway (22).

### miR-379

MiR-379 is differentially regulated in different tumors (i.e., it has been confirmed to be down-regulated in breast cancer and inhibit tumor progression and be over expressed and inhibit cell migration, invasion and EMT in HCC (38). MiR-379-5P functions through targeting focal adhesion kinase (FAK) 3’-UTR, thereby suppressing the AKT pathway. Activating AKT signaling or re-storing FAK expression can alter the tumor suppressing activity of miR-379-5P.

### miR-502-3P

Down-regulation of miR-502-3P was found in both HCC cell lines and tissue samples. Over ex-pression of miR-502-3P dramatically inhibits proliferation, invasion, metastasis and adhesion in HCC. miR-502-3P directly targets SET which is a potent and specific inhibitor of protein phosphatase 2A (PP2A) and is associated with many cellular processes, such as cell cycle control, migration and apoptosis (39, 40). Moreover, miR-502-3p targets protein-coding genes such as SET and thus plays a crucial role in role in carcinoma development especially HCC. Interestingly, downregulation of miR-502-3P induces HCC, while its overexpression inhibits HCC invasion, proliferation, and metastasis, suggesting that targeting this miRNA can be suitable therapeutic option (23).

### miR-193

Many studies have presented the role and importance of miR-193 family in cancer progression, metastasis, and development. The members of this family are miR-193a-3p, miR-193a-5p, miR-193b-3p, and miR-193b-5p, which play crucial role in disease biological processes. mir-193 interacts with different signaling molecules in order to induce suppression of tumor, therefore, it can be used as therapeutic options against HCC in order to make the treatment more effective (41). For instance, MiR-193a-5p has been reported to promote abnormal proliferation and limit apoptosis of HCC cells by targeting the downstream gene BMF, suggesting that miR-193a-5p/BMF axis could be potential therapeutic sites in HCC treatment (42).

### miR-539

MiR-539 acts as a tumor suppressor, which is down-regulated in HCC tissues and cells. Many genes have been shown to be targeted by it including SPAG5, CARMA1, MMP-8, CDK4, and PHB2 (43). A study found that the delivery of miR-539 mimic significantly promoted apoptosis in HCC cells. Studies further showed that miR-539 resulted apoptotic death in HCC cells and tissues. MiR-539 reduced the expression of phosphorylation of STAT3 and anti-apoptotic proteins like Bcl-2 and Bcl-xL. While over-expression of STAT3 considerably reversed the apoptosis mediated by miR-539. Enforced expression of miR-539 eliminated the resistance of HCC cells to arsenic trioxide, which was also confirmed *in vivo* (44).

### miR-613

miR-613 was initially reported to be implicated in lipid metabolism in HepG2 cells and macro-phages (45). miR-613 has been reported to suppress the proliferation and invasion of ovarian can-cer cells and prostate cancer cells (46). Recent studies have shown the link between miR-613 and tumorigenesis. DCLK1 was identified as a novel target of miR-613, which is frequently up-regulated in HCC and associated with tumorigenesis (47, 48). Targeting DCLK1 has been shown to suppress the growth of HCC xenograft tumors in nude mice models (24). Experimental studies revealed that miR-613 has the ability to negatively regulate the expression of DCLK1, which is based on its interaction within the 3′-UTR of DCLK1 (25).

### miR-622

Numerous evidences indicate that miR-622 is frequently down-regulated in human tumors such as gastric cancer, colorectal cancer, pancreatic cancer, and glioma. Interestingly, studies have confirmed that over-expression of miR-622 slow down the growth of HCC xenograft tumors *in vivo*. Bioinformatics analysis and luciferase reporter assay discovered that miR-622 directly targeted the 3′-UTR of mitogen-activated protein 4 kinase 4 (MAP4K4) mRNA. Ectopic expression of miR-622 led to a significant reduction of MAP4K4 expression in HCC cells and xenograft tumors. MAP4K4 over-expression partially counteracted the impact of miR-622 on cell proliferation and apoptosis. Inhibition of JNK and NF-κB signaling simulated the anticancer effects of miR-622 in HCC cells (49).

### miR-885-5p

In 2010, miR-885-5p was identified for the first time from a pheochromocytoma (50). miR-885-5p expression was elevated in patients with cirrhosis or HCC when compared with normal people (51). Both *in vitro* and *in vivo* studies showed that over-expression of miR-885-5p decreased metastasis of HCC cells, and vice versa. Additionally, it was revealed that miR-885-5p suppressed the activity of Wnt/β-catenin signaling pathway by targeting CTNNB1, which proposing miR-885-5p to be a promising negative regulator of HCC development (52).

## Upregulated miRNAs in HCC

### miR-21

miR-21 was significantly up-regulated in HCC tissues and cell lines, which is linked with the in-crease of tumor migration and invasion. The up-regulation of miR-21 can potentially decrease its targeted tumor suppressive factors and therefore promote the development of HCC. Various tar-gets of miR-21 have been experimentally confirmed, including PTEN, PDCD4, RECK, MARCKs, TPM1, and Cdc25A (53, 54). However, ectopic expression of these targets may have diverse functional effects on tumorigenesis. Additionally, it was shown that miR-21 was significantly over-expressed in HCC tissues and cells in expression profiling studies using miRNA microarrays. Inhibition of miR-21 increased the expression of the PTEN and decreased proliferation, migration and invasion of HCC cells.

### miR-27b

miR-27b has been found to play an active role in many kinds of human tumors, which was found to be over-expressed in HCC. MiR-27b over-expression resulted in prominent increased proliferation and reduced apoptosis in Hep3B cells. *In vivo* studies showed that knockdown of miR-27b inhibited the growth of SMMC-7721 cells in mouse xenograft. Furthermore, it was confirmed that Fbxw7 was a direct target of miR-27b.

### miR-24-3p and miR-103

Up-regulation of miR-24-3p and miR-103 was confirmed in HCC tissues, which played an important role in the initiation and progression of HCC by targeting metallothionein 1M (17). AKAP12 is an A-kinase scaffold protein whose down-regulation is linked to an increased risk of tumors including HCC (55). AKAP12 was realized as a tumor suppressor gene because of its capability to suppress growth rates and promote re-organization of the actin based cytoskeleton in v-Src-transformed fibroblasts. miR-103 acts a potential repressor of AKAP12 by directly targeting the 3′-UTR and therefore promotes HCC progression (18). Furthermore, AKAP12 can also binds to key signaling mediators such as PKC, PKA, calmodulin, F-actin, cyclins, Src and phospholipids in a spatiotemporal manner.

### miR-107

*In vitro* and *in vivo* studies have demonstrated that over-expression of miR-107 contributes to proliferation in human HCC. Data showed that miR-107 acts as a tumor promoter in HCC by accelerating growth and metastasis. Notably, CPEB3 was identified as a novel and functional target of miR-107, which acts as a tumor suppressor in HCC. In addition, it was also showed that miR-107 regulates the pathogenesis of HCC partially through the CPEB3/EGFR pathway (16). MiR-107 was involved in a variety of pathological process including carcinogenesis, which was reported to accelerate the proliferation of gastric cancer cells.

### miR-135a

In HCC, miR-135a transcribed by FOXM1 induces the development of portal vein tumor thrombus by inhibiting metastasis suppressor 1 (MTSS1) (56). A study determined miR-135a expression in HCC cells, normal liver cells, HCC tissues and adjacent normal live tissues and examined the effect of miR-135a on cell invasion and migration. miR-135a induced HCC cell metastasis and invasion by targeting the 3′-UTR of FOXO1 mRNA, consequently promoting Snail and MMP2 expression, inhibiting FOXO3a phosphorylation, and promoting AKT phosphorylation (57).

### MiR-155-5p

miR-155-5p and miR-155-3p are different transcripts from the miR-155 host gene, in which miR-155-5p has been considered as the functional form. Previous studies found that miR-155-5p was significantly up-regulated in T cells. Functional manipulation of miR-155-5p expression revealed its important role in regulating Th17 development. Cell proliferation of HCC was enhanced by miR-155-5p both *in vitro* and *in vivo*. FBXW7 was identified as a functional target of miR-155-5p and was involved in the effects of promoting HCC cells proliferation (58).

### miR-519a

miR-519a belongs to the C19MC cluster, which plays a vital role in the pathogenesis of human cancers. Ectopic expression of miR519a increased the cell viability, proliferation and cell cycle progression in HCC cells. PTEN was confirmed to inhibit the progression of HCC by suppressing the PI3k/Akt pathway, and its insufficiency was closely associated with HCC development and progression (59). Studies have shown that the activation of PI3K/Akt signaling pathway pro-motes the progression of HCC and increases the malignant behavior of HCC. MiR-519a pro-motes HCC progression by activating the Akt signaling pathway and inhibiting PTEN (14).

### miR-761

MiR-761 was found to be up-regulated in HCC tissues, which directly target Mitofusin-2 and inhibited its expression. However, a miR-761 inhibitor impaired mitochondrial function and effectively repressed tumor progression *in vitro* and *in vivo* by up-regulating Mitofusin-2.

### miR-765

Abnormal expression of miR-765 frequently occur in a variety of human tumors and contributes to carcinogenesis by affecting various genes, therefore, it was considered as a potential target for cancer diagnosis and therapy. It has been demonstrated that miR-765 is over-expressed in HCC and its expression is correlated with HCC progression. MiR-765 promotes the progression of HCC by targeting and suppressing INPP4B. Suppression of INPP4B up-regulated p-AKT and Cyclin D1, while down-regulated p-FOXO3a and p21 in HCC. From these findings it was revealed that miR-765 acts as a potential onco-miR. INPP4B acts a tumor suppressor by regulating PI3K/Akt signaling pathway (13).

### miR-935

miR-935 over-expression promotes cell proliferation, tumorigenesis and cell cycle progression, and vice versa. MiR-935 down-regulates the expression of sex determining region Y-Box 7 (SOX7) and increases C-Myc and cyclin D1, two G1/S transitional regulators (60). Certain evidences have shown that SOX7 may disrupt the transcriptional function of the β-catenin-TCF/LEF interaction and inhibit the activity of Wnt target genes including cyclin D1, c-Myc and COX-219. Several recent studies have shown that SOX7 acts as a tumor suppressor through the Wnt/β-catenin signaling pathway in various cancers (61).

### miR-1180

Over-expression of miR-1180 promotes the proliferation of HCC by directly suppressing TNIP2, then it can up-regulate the NF-kB downstream target genes including cy-clin D1, Myc and p-Rb. Generally, it is accepted that TNIP2 played an essential role in the regulation of NF-kB pathway, which act as a transcription factor that facilitates a number of biological processes including cell proliferation. Previous studies have indicated that upregulation of miR-1180-3p induces HCC and is associated with poor prognosis, suggesting that it might be useful as a prognostic marker in the case of HCC. Moreover, targeting miR-1180 through inhibitors can decrease the risk of HCC or alleviate HCC progression and metastasis (62, 63).

### miR-4262

MiR-4262 is over-expressed in HCC tissues and cell lines. Results showed that miR-4262 promotes cell proliferation by suppressing PDCD4 and promotes the accumulation of nuclear NF-kB/P65 by activating NF-kB pathway (11). PDCD4 functions as a tumor suppressor which regulates both transcription and translation in many cancers. It was confirmed that PDCD4 inhibits NF-kB dependent transcription in human glioblastoma cells by its direct interaction with p65 (64).

## Gene Ontology and KEGG Analysis

**Figure 3:**
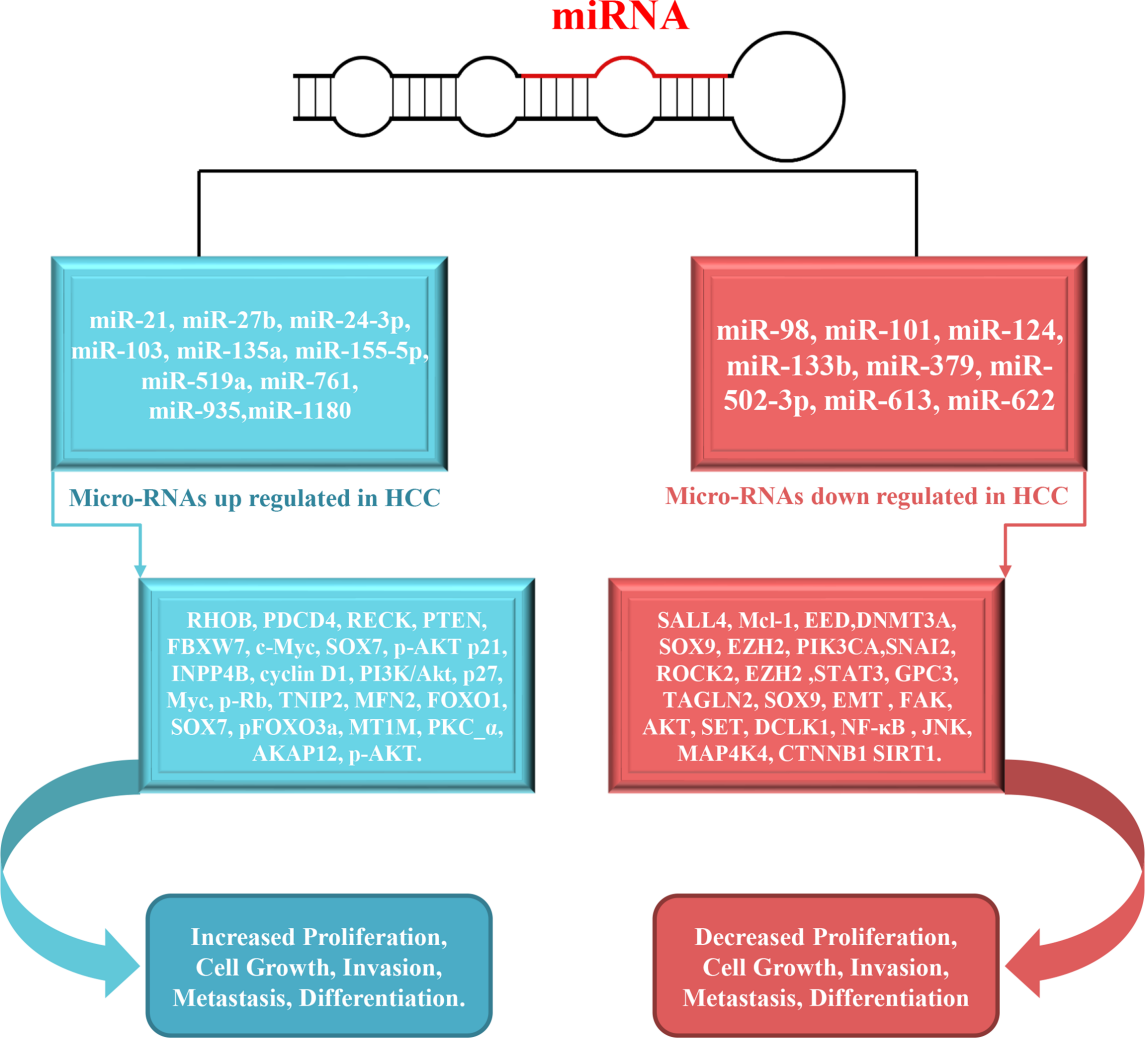
Schematic overview of miRNAs involved in the metastasis, proliferation, migration and invasion in HCC. The inhibition of validated targets (left side) by specific miRNAs results in increased metastasis, proliferation, migration and invasion.

**Figure 4:**
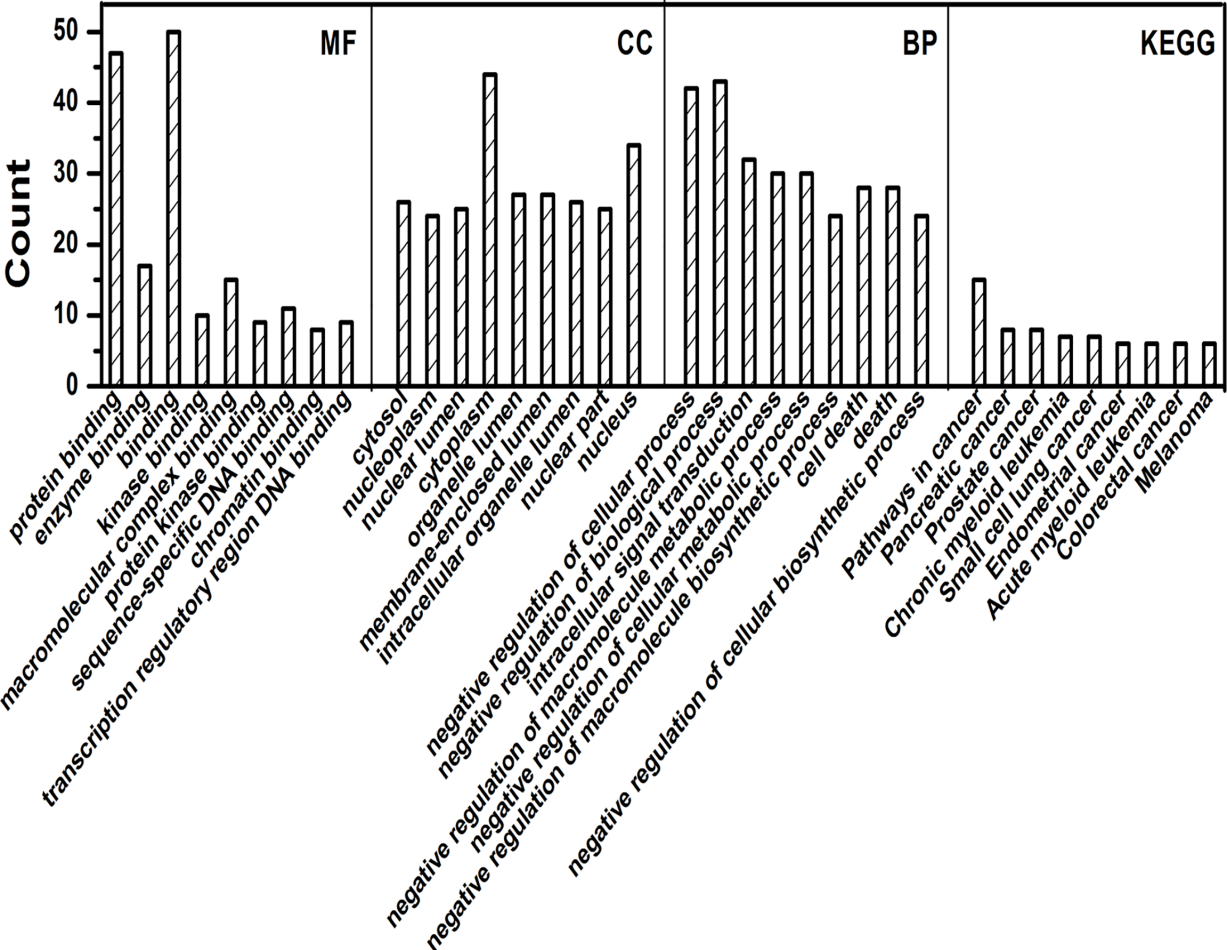
GO and KEGG analysis by R package cluster Profiler. Enrichment analysis of Gene Ontology for targeted genes that include biological processes (BP), molecular function (MF), and cell fractions (CC) as well as KEGG pathways analysis.

Gene ontology and KEGG analysis was carried out for a number of genes. GO analysis showed that gene annotations are mainly in three aspects: biological processes (BP), molecular function (MF) and components (CC). In the biological process, the genes were mainly enriched in negative regulation of cellular process, intracellular signal transduction, negative regulation of macromolecule metabolic process, negative regulation of biological process and many more as shown in **Figure 3**. In the molecular function, the genes were mainly involved in protein binding, enzyme binding, molecular complex binding and other molecular functions. In the cell components, the expressed genes were mainly found in cytoplasm, nucleus, cytosol, organelle lumen, membrane enclosed lumen, intracellular organelle lumen and other cell components. These data showed that the genes exhibited different GO functions, implying the different roles of gene. In KEGG analysis, the genes were mainly involved in a number of pathways linked with pancreatic cancer, small cell lung cancer, prostate cancer, colorectal cancer and many other malignancies as shown in **Figure 4**.

## miRNAs Confirmed as Potential Therapeutic Targets and Diagnostic Markers in HCC

Differential and specific biomarkers are crucial for early detection and diagnosis of HCC, as well as the advancement of preventive screening. Nonetheless, current strategies are inadequate for detecting HCC in early stage. Advance in MRI and CT have incredibly improved the diagnostic efficacy of HCC. Serum alfa-fetoprotein (AFP) and des-gamma-carboxy prothrombin (DCP) levels have been utilized as HCC biomarkers. However, the exactness of AFP is modest with a sensitivity 39%– 65% and specificity 76%– 94%. However, 1/3 of HCC patients got missed diagnosis based on AFP and AFP levels can also be nonspecifically increased in patients with hepatitis and cirrhosis (65).

Numerous miRNAs are dysregulated in HCC and circulating miRNA levels are additionally influenced by HCC progression. It is fascinating that circulating miR-21, miR-222, and miR-223 were observed to be up-regulated in HCC patients with HBV or HCV. Circulating miR-21 ex-pression was significantly higher in HCC patients than in those with perpetual hepatitis and healthy control. Receiver operating characteristic (ROC) investigation of miR-21 yielded an Area Under Curve (AUC) of 0.773 while separating HCC from perpetual hepatitis, and an AUC of 0.953 when separating HCC from healthy control. The values of miR-21 were better than AFP as biomarker in HCC. Meantime, serum levels of miR-1, miR-25, miR-92a, miR-206, miR-375, and miR let-7f were elevated in HCC patients.

miR-130b had the biggest AUC of 0.913, besides that, with an affectability of 87.7% and a specificity of 81.4% for distinguishing HCC. miR-15b had the affectability of 98.3% for identifying HCC, which is the most astounding one in all the miRNAs analyzed, despite its low specificity of 15.3%. The high affectability of miR-15b and miR-130b as HCC biomarkers may ensure patients with early stage HCC better prognosis. Similarly, serum miR-16 level was observed to be a more sensitive biomarker for HCC than AFP and DCP. The combination of miR-16, AFP, AFP-L3% and DCP yielded the optimum state of both affectability (92.4%) and specificity (78.5%) for HCC diagnosis, when investigation was limited to patients with tumors less than 3 cm. Moreover, a current meta-analysis in which eight investigations were incorporated demonstrated that the diagnostic value of miRNAs for HCC as follows: pooled affectability 0.87 (0.72–0.98), pooled specificity 0.90 (0.76–1.00), pooled positive probability proportion 8.7 (3.52– 97.45), pooled negative probability proportion 0.13 (0.02– 0.31), and pooled indicative chances proportion 86.69 (19.06– 2,646.00) (66).

miR-122, miR-125b, and miR-192 in serum are potential non-invasive biomarkers for identifying liver damage in patients. miR-122 act as a solid marker particularly to detect hepatitis B. Besides, when contrasted with ALT, miR-122 was found to reflect liver injury with a better AUC of 0.784. These discoveries recommended the capability of utilizing miR-122 as a delicate and enlightening biomarker for hepatitis B. In addition, miR-125b could be possibly utilized as a biomarker for HCV and miR-192 for drug-induced liver injury. The affectability and specificity of utilizing a single biomarker and combined biomarkers for liver disease has been investigated. The most extreme value of Youden’s index come about after combination of miR-122 and miR-125b, in which affectability increased from73.41% to 83.89%, and specificity diminished from 83.05% to76.27%. Studies showed that the serum level of miR-122 was connected with ALT in HBV patients and even varied more sensitively than ALT (67). In this manner, miR-122 could be utilized as a more precise biomarker for HBV-related liver injury than ALT, a traditional biomarker of liver damage. This increased levels of miR-122 and miR-125b are related with less progression and better survival of HBV-induced liver injury patients. In general, the study provides clinical confirmation of circulating miR-122 as a biomarker for HBV patients, as well as the possibility of miR-125b and miR-192 as biomarkers for HCV and chemical related liver damage. Moreover, the combination of miR-122 and miR-125 demonstrated a higher affectability than ALT. Even though the affectability and strength of miRNAs as biomarkers for liver disease was reasonable, proper controls should be utilized. While evaluating the specificity of a miRNA for detecting HCC, it is important to make sure that examiners should be coordinated by age, sex, etiology and severity of illness.

## miRNA Act as Tumor Suppressor

miR-493 was considered as a tumor suppressor in different human tumors by targeting on various molecules. In human bladder cancer cells, miR-493 reduces cell motility and movement capacity by targeting FZD4 and Rho C. miR-493 can also suppress tumor development, invasion and metastasis of lung cancer by regulating ERK, E2F1 and PI3K-AKT pathway. IGF1R and MKK7 were distinguished as immediate targets of miR-493, which suppress liver metastasis of colon tumor cells. miR-493 may stifle the tumorigenesis of HCC by modulating Wnt signaling path-way. While miR-493 is significantly down-regulated in HCC patients, restore of miR-493 could remarkably suppress the proliferation, invasion, migration and tumorigenicity of HCC cells *in vitro* and *in vivo*. miR-493 could down-regulate ANTXR1 and RSPO2 specifically and Wnt/b-catenin pathway was engaged with its anti-tumor capacity.

## miRNAs Act as Therapeutic Targets

Recently, accumulated evidences have demonstrated that methodologies focused on regulation of miRNA expression could be a novel approach to malignancy treatment. Previous studies have shown that inhibition of miR-122 by modification of miRNA oligonucleotides was a promising strategy for minimizing miRNA action in non-human primates (68). Another investigation has exhibited that restoration of tumor suppressor miR-122 makes HCC cells more sensitive to sorafenib treatment by down-regulating of multidrug protection genes. Alternatively, suppression of oncogenic miR-221 brought about better survival and significant decrease in the number and size of tumors. Besides, HCC cells transfected with anti-miR-221 were more sensitive to chemo-therapy with joined interferon-α and 5-FU.

## Conclusion and Future Perspective

miRNAs play an important role in patho-physiological processes of the initiation and progression of HCC. Many miRNAs are abnormally expressed and play a vital role in HCC progression by specifically targeting at critical genes. Various reports have identified potential miRNA biomarkers, their target genes and the possible mechanisms that lead to hepatocarcinogenesis and advancement. MDR is a profoundly dilemma in tumor treatment process, which was realized to be regulated by a wide range of miRNAs. Along these lines, miRNAs could be utilized as ideal biomarkers for predicting chemotherapeutic reaction and potential targets in HCC treatment. For instance, miR-98 targets SALL4, miR-124 targets STAT3, miR-502-3P targets SET, miR-761 targets MFN2 and miR-222 targets P27. Among those some miRNAs even have multiple target genes that are involved in HCC progression. For example, miR-221 and miR-222 target p27, p57, Bmf, PTEN, TIMP3, DDIT4 and PPP2R2A. While miR-122 targets cyclin G1, ADAM10, AD-AM17, SRF, Igf1R and Bcl-w. As contrast, one target gene can be regulated by multiple miR-NAs simultaneously. These findings show that deregulated miRNAs and their targets make up a complex interacting network, which influence HCC development and progression. Future re-search is needed to address and expand the therapeutic potential of miRNAs in inhibiting the progression of HCC. In addition, miRNAs may provide new perspectives and targets for the development and research of novel HCC treatment modalities.

## Author Contributions

Study concept and design: SK, QB, MF. Manuscript writing: SK, D-YZ, MH, JR, SN. Analysis and interpretation of data: MF, J-YZ, JR, SN. Acquisition of data: D-YZ, MH, JR, SN. Critical revision of manuscript and video: SK, QB, MF. Funding obtained: SK, QB, MF. All authors contributed to the article and approved the submitted version.

## Funding

This study was financially supported by grants from Henan province innovation talents of science and technology plan (No. SB201901045) and hepatobiliary foundation of Henan Charity General Federation (No: GDXZ2019006).

## Conflict of Interest

The authors declare that the research was conducted in the absence of any commercial or financial relationships that could be construed as a potential conflict of interest.

## Publisher’s Note

All claims expressed in this article are solely those of the authors and do not necessarily represent those of their affiliated organizations, or those of the publisher, the editors and the reviewers. Any product that may be evaluated in this article, or claim that may be made by its manufacturer, is not guaranteed or endorsed by the publisher.
